# Advances in understanding of dendritic cell in the pathogenesis of acute kidney injury

**DOI:** 10.3389/fimmu.2024.1294807

**Published:** 2024-02-16

**Authors:** Dongfang Lv, Huihui Jiang, Xianzhen Yang, Yi Li, Weipin Niu, Denglu Zhang

**Affiliations:** ^1^ College of First Clinical Medicine, Shandong University of Traditional Chinese Medicine, Jinan, China; ^2^ Clinical Laboratory, Affiliated Hospital of Shandong University of Traditional Chinese Medicine, Jinan, China; ^3^ Department of Urology, Afliated Hospital of Shandong University of Traditional Chinese Medicine, Jinan, China; ^4^ Department of Central Laboratory, Shandong Provincial Hospital Affiliated to Shandong First Medical University, Jinan, China; ^5^ Engineering Laboratory of Urinary Organ and Functional Reconstruction of Shandong Province, Shandong Provincial Hospital Affiliated to Shandong First Medical University, Jinan, China; ^6^ Central Laboratory, Affiliated Hospital of Shandong University of Traditional Chinese Medicine, Jinan, China; ^7^ Shandong Key Laboratory of Dominant Diseases of traditional Chinese Medicine, Affiliated Hospital of Shandong University of Traditional Chinese Medicine, Jinan, China

**Keywords:** dendritic cells, acute kidney injury, protective proinflammatory, tolerogenic immune reactions, tolerogenic dendritic cells

## Abstract

Acute kidney injury (AKI) is characterized by a rapid decline in renal function and is associated with a high morbidity and mortality rate. At present, the underlying mechanisms of AKI remain incompletely understood. Immune disorder is a prominent feature of AKI, and dendritic cells (DCs) play a pivotal role in orchestrating both innate and adaptive immune responses, including the induction of protective proinflammatory and tolerogenic immune reactions. Emerging evidence suggests that DCs play a critical role in the initiation and development of AKI. This paper aimed to conduct a comprehensive review and analysis of the role of DCs in the progression of AKI and elucidate the underlying molecular mechanism. The ultimate objective was to offer valuable insights and guidance for the treatment of AKI.

## 1 Introduction

Acute kidney injury (AKI) is defined as a sudden decrease in the glomerular filtration rate, as evidenced by a 50% increase in serum creatinine (SCr) within 7 days, a 0.3 mg/dL increase in SCr within 2 days, or oliguria (1). In recent years, the incidence of AKI due to chronic kidney disease has been increasing due to the ageing of the population and the increasing prevalence of underlying conditions such as diabetes mellitus and hypertension (2). Furthermore, in conjunction with the continued emergence of novel pharmaceuticals and the use of interventional therapies, drug-induced AKI is increasingly contributing to the aetiology of this condition (3). A global meta-analysis of AKI prevalence showed that the incidence of AKI was 21.6% among adults and 33.7% among children, and AKI-related mortality rates were 23.9% among adults and 13.8% among children (4). Due to the increased rates of morbidity and the high rate of mortality, AKI has become a significant public health concern in contemporary society, and it represents a challenging and prominent area of current research.

The aetiology of AKI is multifactorial and includes renal ischaemia, nephrotoxin exposure, and sepsis, each with distinct pathophysiological mechanisms (5). During the progression of AKI, the inflammatory response plays a crucial role, and the innate and adaptive immune systems participate in the inflammatory process. Throughout the progression of AKI, various factors contribute to the activation and recruitment of immune cells to the injured kidney, including damage-associated molecular patterns (DAMPs), hypoxia-inducible factors (HIFs), renal vascular endothelial dysfunction, adhesion molecules, chemokines, cytokines, and Toll-like receptors (6). Dendritic cells (DCs), neutrophils, macrophages, and lymphocytes are immune cells that are implicated in the pathophysiology of AKI, and some of their subsets are involved in healing processes (7).

In terms of innate and adaptive immune responses, DCs are specialized antigen-presenting cells (APCs). DCs constantly sense pathogen- or inflammation-associated signals and keep peripheral T cells in a resting state in the absence of DAMPs or inflammation (8). Once DAMPs or inflammation-related signals are recognized by pattern recognition receptors (PRRs) on DCs, DCs become activated or mature and migrate toward the injured tissue (9). Mature DCs can efficiently ingest antigens, process them into proteolytic peptides, and load these peptides onto major histocompatibility complex (MHC) class I and class II molecules to form MHC-peptide complexes (10). DCs then migrate from the site of antigen uptake to secondary lymphoid organs and can present antigens to CD8^+^ T cells and CD4^+^ T cells, inducing effector T cell differentiation and regulatory T (Treg) cell tolerance, thereby initiating an antigen-specific immune response or immune tolerance (11, 12). DCs also secrete cytokines and growth factors enhance or regulate the immune response. Because immune cells are recruited to the site of injury after the onset of AKI and DCs are efficient APCs, in this paper, we examined the role of DCs during the onset of AKI and the recent research advances in this area of study.

## 2 Subsets and ontogeny of DCs

**Figure 1:**
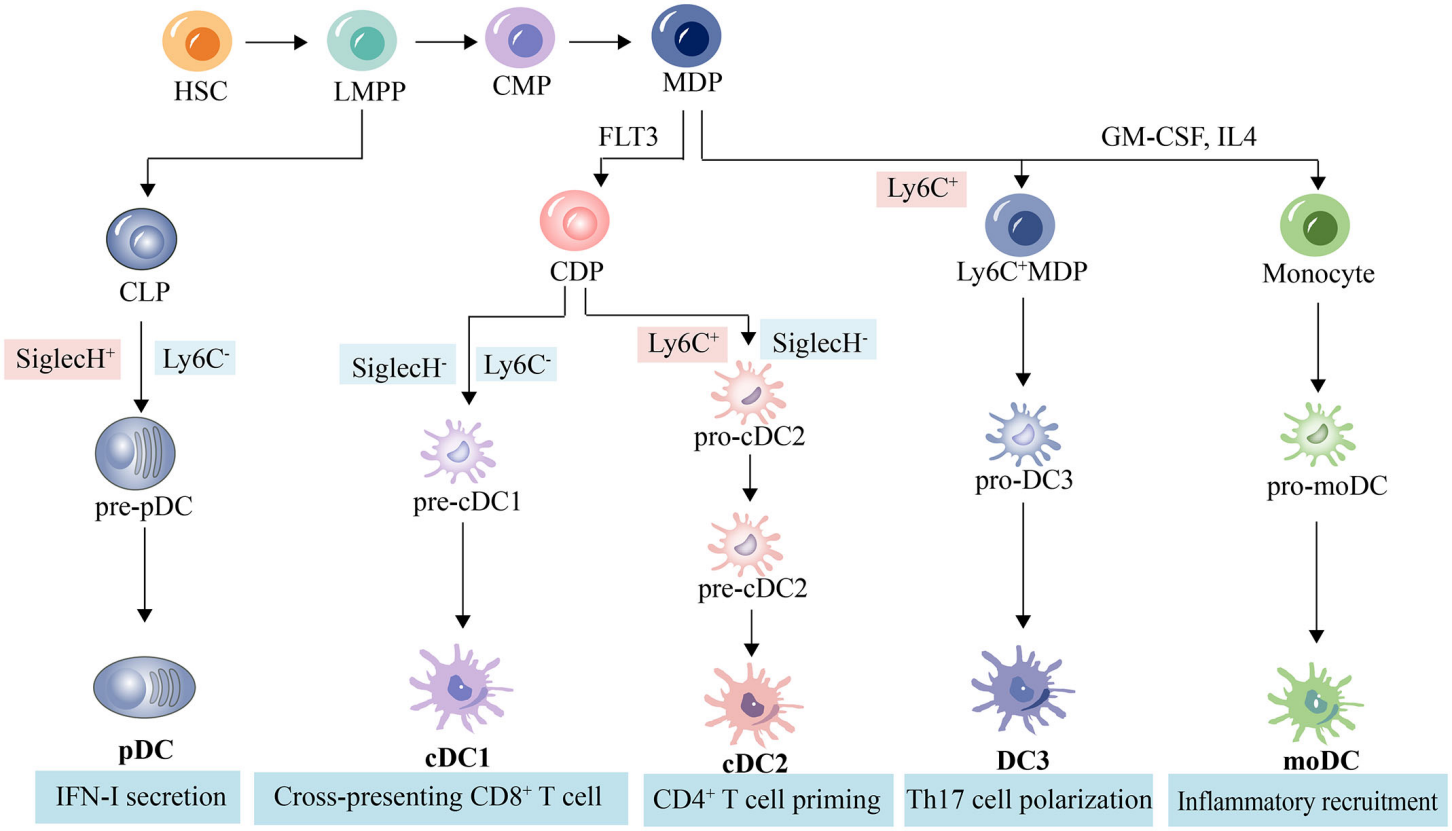
DC subpopulations and related lineages in mice. Hematopoietic stem cells differentiate into pluripotent progenitors (LMPP), which further differentiate into common myeloid progenitors (CPM) and common lymphoid progenitor (CLP). CLP then differentiate into pDC precursors and ultimately pDC. CPM differentiate into macrophage dendritic progenitors (MDP), which in turn differentiate into common DC progenitors (CDP), Ly6C^+^MDP, and monocytes. cDP give rise to cDC precursors, which are induced by FLT3 and ultimately give rise to cDC or pDC (39, 40). Monocytes produce moDC from granulocyte-macrophage colony-stimulating factor (GM-CSF)- and interleukin-4 (IL-4)-stimulated myeloid stem cells (41, 42). Ly6C^+^MDP produce pro-DC3s and eventually DC3s (23).

**Figure 2:**
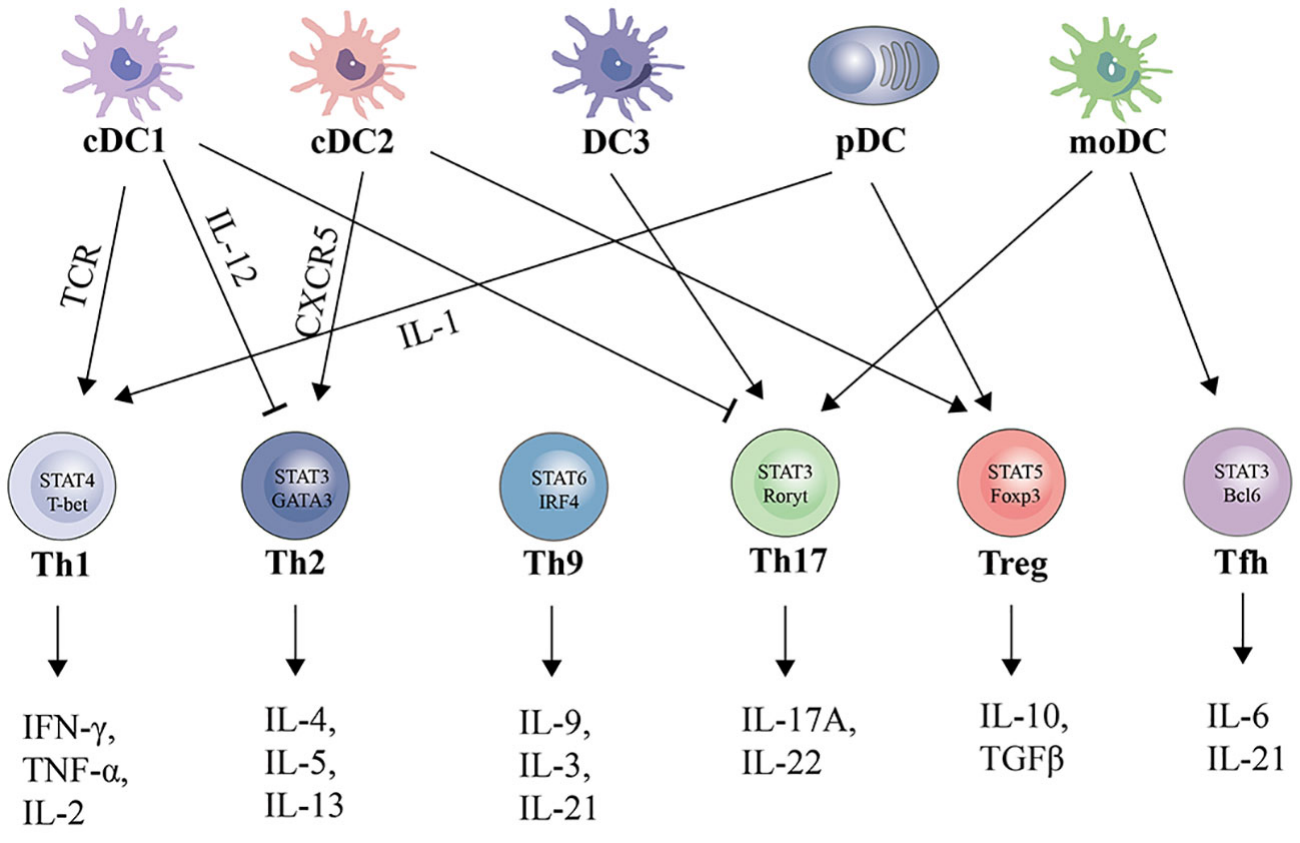
Subsets, polarizing cytokines, and effector cytokines of DCs-regulated CD4^+^ cell polarization.

DCs can be categorized into different subsets, and different subsets have significant phenotypic heterogeneity and functional plasticity. The terminology used to describe these different subsets has changed in recent years. More recently, consistency in assigning subsets has been adopted by many groups based on origin, associated regulatory transcription factors, surface markers, and biological function (13, 14). We briefly summarize the basic characteristics of different subsets of DCs, including conventional DCs (cDCs), plasmacytoid DCs (pDCs), monocyte-derived DCs (mo-DCs), and newly discovered DC3s as shown in **Table 1**; **Figure 1**. The mechanism of DC-induced helper T cell polarization for different subsets of DCs is shown in **Figure 2**.

**Table 1:** Key transcription factors, markers and cytokines ofDC subsets.

Subsets	Key transcription factors	Key markers		Cytokines	Refs
		Mouse	Human		
cDC1s	Batf3, Irf8, Id2, Nfil3	CLEC9A^+^, XCR1^+^, CD103^+^, CD26^+^, CD8^+^	CD141^+^, CD14^-^, CLEC9A^+^, XCR1^+^, FLT3^+^, CD103^+^	IL-12, IFN-III	(15–18)
cDC2s	Relb, Rbpj, Irf4	, CD26^+^, CX3CR1^+^, CD11b^+^, F4/80^-^	CD1c^+^, CD5^+^, CD14^−^, CD163^−^	IL-6, IL-23	(19, 20)
DC3s	IRF8, Klf4	CD172a^+^, Lyz2^+^, CD16/32^+^	CD5-, CD163+, CD14+	/	(21–24)
pDCs	E2-2, Tcf4, Irf8, Irf7, Stat3, Stat5	CD11c^-^, MHC II^-^, CD11b^−^, B220^+^, CD123^+^, Ly6C^+^, BST2^+^, SiglecH^+^	BDCA2^+^, CD11c^−^, CD4^+^, BDCA4^+^, CD123^+^	IFN-α	(25–27)
moDCs	PU.1, IRF4, NR4A3, NCOR2, Etv3, Etv6	, CX3CR1^+^, CD11b^+^, F4/80^+^, Ly6C^+^	CD86^+^, CD40^+^, CD80^+^, CD83^+^, CCR7^+^, CD14^-^, CD209^+^	TNF-α, IL-1, IL-12, IL-23, iNOS	(28–33)
tolDCs	Nrf2, Baff	CD40^-^, CD80^-^, CD86^-^, MHC-II^-^, PD-L1^+^	CD163^+^, CD141^+^, CD16^+^, CD14^+^	IL-10, TGF-β, IFN-γ	(34–38)

### 2.1 cDCs

cDCs are distributed in almost all lymphoid and nonlymphoid tissues and are divided into two subgroups, cDC1s and cDC2s. cDCs serve as the principal APCs in the immune system and can detect tissue damage and effectively capture and process environmentally associated and cell-associated antigens. Subsequently, cDCs transport these antigens to the draining lymph nodes for presentation to T cells (43).

cDC1s specialize in the antigenic cross-presentation to CD8^+^ T cells, the activation of T helper type 1 (Th1) CD4^+^ T cells, and the generation of type III interferons (IFN-III) (44). Murine cDC1s are typically characterized as CLEC9A^+^, XCR1^+^, CD103^+^, CD26^+^, and CD8^+^ (15). Human cDC1s are characterized as CD141^+^, CD14^-^, CLEC9A^+^, XCR1^+^, FLT3^+^, and CD103^+^ (19). Almost all human and mouse cDC1s express XCR1^+^, Clec9A^+^, and CD103^+^. Developmentally, cDC1s depend on transcription factors Batf3, Irf8, Id2, and Nfil3 (16–18).

cDC2s primarily facilitate the differentiation of T helper type 2 and type 17 (Th2 and Th17) CD4^+^ T cells and the secretion of interleukin-6 (IL-6) and interleukin-23 (IL-23) (45, 46). The development of cDC2s is largely dependent on Relb, Rbpj, Irf4 (19, 20). Murine cDC2s are typically characterized as CD26^+^, CX3CR1^+^, CD11b^+^, F4/80^-^, and in human as CD1c^+^, CD5^+^, CD14^−^, CD163^−^ (45). In addition, the expression of SiglecH and Ly6C distinguishes precursor cells that will become cDC1s (SiglecH^-^; Ly6C^-^), cDC2s (SiglecH^-^; Ly6C^+^) and pDCs (SiglecH^+^; Ly6C^-^) (47). Notably, Irf8 deficiency induces the transcriptional, functional, and epigenetic reprogramming of cDC1s to cDC2s (48).

### 2.2 pDCs

pDCs are a unique sentinel cell type with a plasma cell-like capacity for rapid and high production of interferon type I (IFN-I) in response to viruses, as well as the ability to differentiate into cDCs (49). Upon activation of human plasma cell-like predendritic cells (pDCs) by a single microbial or cytokine stimulus, the cells differentiated into three stable subpopulations (P1-P3), and notably the pDCs differentiated into subpopulations after SARS-CoV-2 infection and rapidly produced IFN-I and IFN-III upon viral exposure (50, 51). Murine cDC1s are typically characterized as CD11c^-^, MHC II^-^, CD11b^−^, B220_+_, and CD123^+^ (52). Human cDC1s are characterized as BDCA2^+^, BDCA4^+^, CD11c^−^, CD4^+^, and CD123^+^ (53). pDCs develop primarily through an Flt3-driven pathway shared with cDCs, and the transcription factor PU.1 controls Flt3, while subsequent specialization of pDCs requires the helix-loop-helix transcription factor (E protein) E2-2/Tcf4 (25, 26). E2-2/Tcf2-deficient pDCs are converted to cDCs *in vitro*. E2-2 deficiency is associated with aberrant expression profiles and impaired IFN responses in pDCs, and E2-2 directly activates transcription factors (SpiB, Irf8) and functional factors (Irf7) involved in pDC development (54). Furthermore, Stat3 is required for DC progenitor cell expansion, whereas Stat5 inhibits the transcriptional network of pDCs in Irf8 and lineage-negative, Flt3+ progenitor cells to control pDC production (27).

### 2.3 moDCs

moDCs is a subset of DCs formed by monocytes during inflammation.Monocyte-derived cells exhibit high plasticity to the environment and show high susceptibility to inflammatory stimuli in an inflammatory microenvironment, including increased levels of CCL2 and IL-8, which promote monocyte recruitment (55). Monocytes are stimulated by GM-CSF and IL-4 to form immature moDCs, which differentiate into mature moDCs when stimulated by inflammatory cytokines or PAMPs (56). Fully differentiated moDCs acquire DC morphology and localize to T-cell regions via L-selectin and CCR7 (57). Increased production of inflammatory factors and chemokines, such as TNF-α, IL-1, IL-12, IL-23 and CXCL10, was observed in LPS-stimulated GM-CSF-induced bone marrow-derived moDCs (28, 29). In contrast to the increased antigen-presentation and migratory capacity of cDCs, moDCs mainly coordinate the secretion of inflammatory factors and chemokines for local immune responses. The transcriptional factors PU.1, IRF4, NR4A3, NCOR2, Etv3 and Etv6 control the differentiation of human monocytes into moDCs, and the production of moDCs can be inhibited by targeted modulation of these transcription factors, which provides a new perspective on inflammatory diseases (30–33).

### 2.4 DC3s

DC3s are becoming better known as a new subset of DCs, and a developmental atlas of DC3s has been established (21). Human DC3s were identified as CD5^-^CD163^+^CD14^+^ cells (22). Murine DC3s were identified as CD172a^+^Lyz2^+^CD16/32^+^ cells derived from Ly6C^+^ monocyte-dendritic cell precursors (23). Irf8 is a key transcription factor that regulates the development and maintenance of DC3s, and patients with low Irf8 expression show reduced cDC and pDC, while DC3 is maintained or amplified (24). In addition, the transcription factor Klf4 is also involved in regulating DC3s development, and Klf4 deficiency affects the transition of Ly6C+ MDPs to pro-DC3s (23). Functionally, DC3s have the ability to induce the transformation of T cells into IL-17A-producing T helper-17 (Th17) cells, which makes DC3s important players in inflammatory diseases and immune regulation (22).

## 3 Kidney DCs (kDCs) in homeostasis

In a state of renal health, kDCs act as sentinels, using their dendrites to continuously monitor the kidney milieu (58–60). kDCs sample autoantigens from the tubules and glomeruli and subsequently migrate to the renal lymph nodes (61, 62). Within these lymph nodes, kDCs maintain immune self-tolerance, immune system functionality, and tissue equilibrium by inducing the activation of Treg cells, restraining T-cell activation, proliferation, and effector capacities, and accommodating autoreactive T cells in the presence of T helper cells (13, 63). Furthermore, kDCs play a crucial role in fostering immune tolerance toward harmless antigens present in the circulation. Specifically, low molecular weight antigens are concentrated and sieved within the kidney, subsequently reaching the kidney lymph nodes through lymphatic drainage. Within these lymph nodes, kDCs can capture these antigens and induce apoptosis in cytotoxic T cells by means of PD-L1 expression (62, 64). In a state of homeostasis, the movement of DCs into lymph nodes to execute these functions is regulated by the expression of CCR7 (65). The passage of low molecular weight antigens through this tolerance mechanism plays a significant role in the prevention of undesired immune responses, and kDCs play a role in maintaining peripheral immune tolerance to these harmless antigens. Notably, this tolerance mechanism is disrupted in diseases characterized by proteinuria. In these diseases, the glomerular filters become permeable, leading to increased recognition and acceptance of filtered proteins by kDCs, including high molecular weight proteins. Consequently, this stimulates potentially harmful T cells, providing an additional mechanism through which proteinuria can cause damage (60). The characteristics and functions of monocyte-derived tolerant DCs and mature DCs are shown in **Table 2**.

**Table 2:** The induction factors, secretion factors, phenotypes and functions of tolerogenic DCs and mature DCs.

	Induction factors	Phenotypes	Cytokines	Functions	Refs
Tolerogenic DCs	IL-10, TGFβ, VIP, IFN-γ, Galectin-1, Vitamine D3, Ra	CD40^-^, CD80^-^, CD86^-^, MHC-II^-^, PD-L1^+^	IL-10, TGFβ	Treg cell activation	(66)
Mature DCs	LPS, TNF, IL-1β, PGE2, IL-6	CD80+, CD83+, CD86+, CD40+, MHC-II+	TGF- α, IL-1β, IL-6, IL-12	Induction of effector/cytotoxic T cell respons	(67)

## 4 kDC maturation and migration in AKI

As specialized APCs, DCs migrate to the kidney, which is critical for initiating protective proinflammatory and tolerogenic immune responses in AKI. The transport of different DC subpopulations in the kidney and renal lymph nodes is essential for DC-dependent activation and modulation of inflammation and immunity. DC chemotaxis and migration are triggered by interactions between chemokines and their receptors (68). After the onset of AKI, renal DCs are activated, increase in number and activity and have an enhanced ability to present antigens to T cells in renal draining lymph nodes (69). Previous studies have shown that CCR and CC chemokine expression is closely associated with FLT3 ligand-induced migration of renal DCs, including CCR1, CCR2, CCR5, CX3CR1, CCR7, CCL19, and CCL21 (59, 61). cDCs, which are the major DC type, mature in response to stimulation with DAMPs or inflammatory signals and express high levels of CCR7, which interacts with its ligands CCL19 and CCL21 to direct mature cDCs transport to the lymph nodes via afferent lymphatic vessels to regulate T-cell immunity (70–72). After ischaemic kidney injury, dilated renal lymphatic vessels express high levels of CCL21, which stimulates the recruitment of more CCR7^+^ DCs to renal draining lymph nodes, worsening renal inflammation and fibrosis, and inhibiting CCR7 expression (blocking the binding of VEGF-C/D to VEGFR3) or renal lymphangiogenesis reduces the migration of CCR7^+^ DCs (73). Furthermore, CCR7 and its ligand SLC/CCL21 are constitutively expressed in glomeruli in adjacent cell types in the human kidney and play a role in glomerular homeostasis and regenerative processes (74, 75).

**Figure 3:**
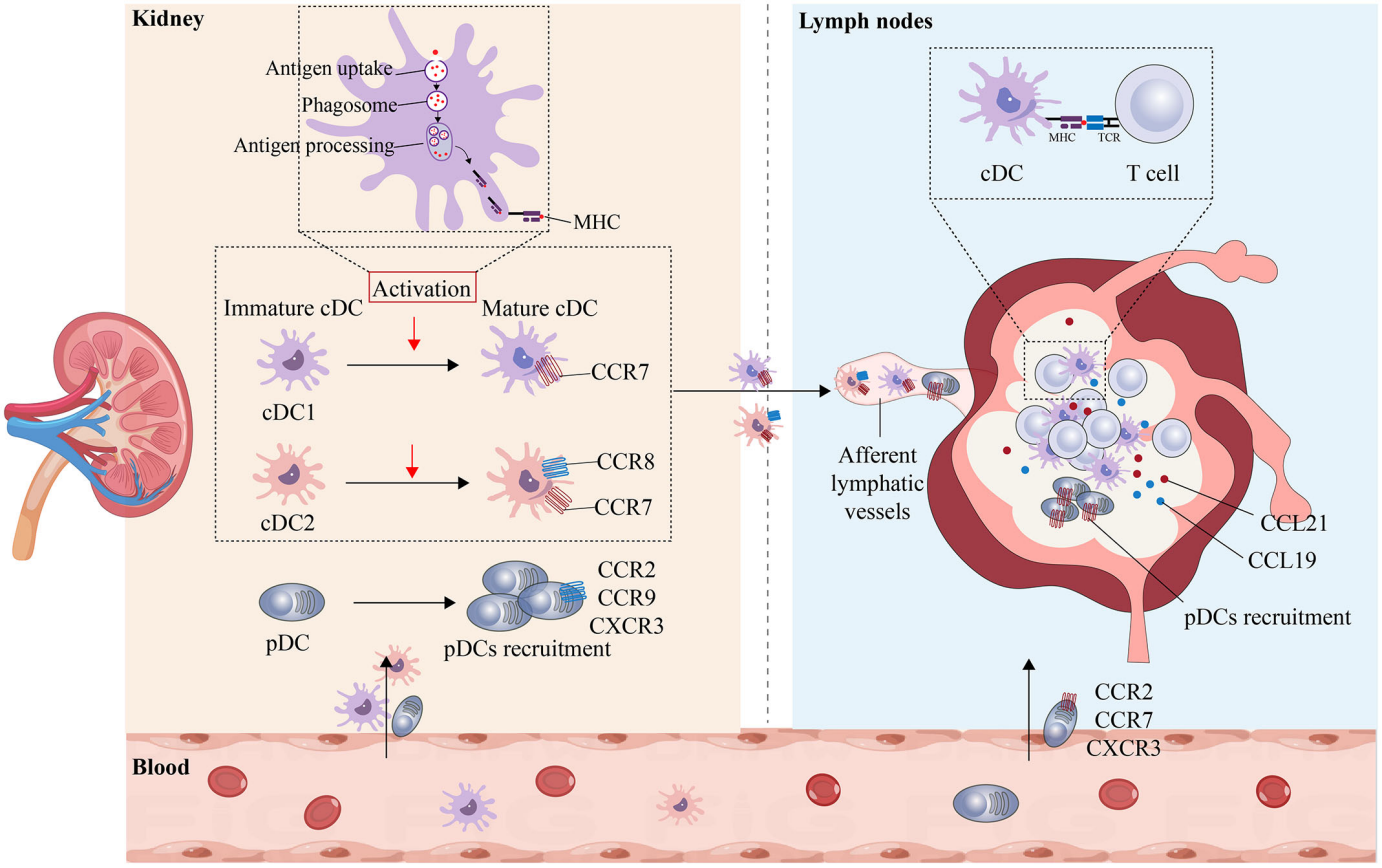
Maturation and migration of kDCs in AKI. The ability of DCs in the peripheral blood to migrate to the kidneys plays an important role in maintaining health and mediating disease. In the healthy state, DCs that enter the kidney are in an immature state and maintain renal immune tolerance to innocuous antigens. In AKI, immature cDCs are activated and undergo maturation, resulting in the upregulation of MHC and costimulatory molecules, the uptake and processing of antigens, and an increase in CCR7 expression. Mature cDCs can then migrate toward the lumen of lymphatic vessels containing CCL19 and CCL21, where they bind to T cells and present antigens to T cells. In the inflammatory state, pDCs express CCR2, CCR9, and CXCR3, are recruited from the peripheral blood to the site of injury and can directly enter the lymphatic lumen by expressing CCR2, CCR7, and CXCR3.

Under normal physiological conditions, pDCs are mainly located in the peripheral blood and T-cell-rich lymphoid tissues. Under pathological conditions, pDCs migrate from the peripheral blood into inflamed tissues and lymph nodes to initiate an immune response (76). Multiple chemokine receptors are highly expressed on the surface of pDCs, and the chemotaxis of pDCs is promoted by the binding of corresponding ligands; for example, when CCR2, CCR9, and CXCR3 receptors are expressed on the surface of pDCs, the transfer of pDCs from the peripheral blood to inflammatory tissues is promoted, and when CCR2, CCR7, and CXCR3 receptors are expressed on the surface of pDCs, the direct transfer of pDCs from the peripheral blood to the lymph nodes is promoted (68). While the chemokines responsible for regulating the recruitment and migration of pDCs in various tissues, such as the small intestine (77), have been confirmed, the precise factors governing pDC recruitment in AKI remain unclear. The regulatory mechanisms by which DCs are activated and mature and their migration to the site of renal injury and renal lymphatics are shown in **Figure 3**.

## 5 Mechanisms of the kDC-mediated immune response in AKI

### 5.1 Renal ischaemia–reperfusion injury (RIRI)

**Figure 4:**
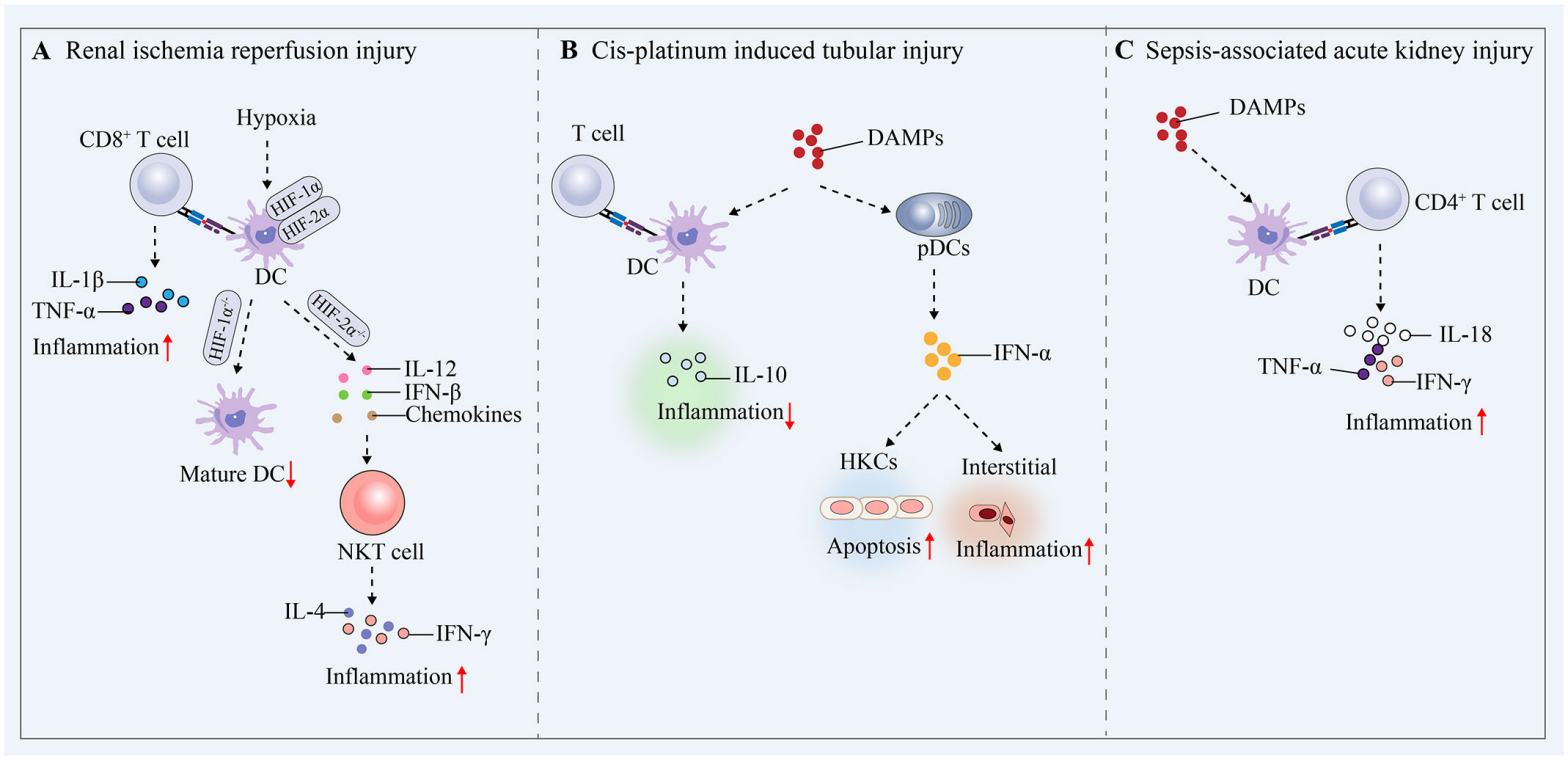
Mechanisms by which DCs mediate protective proinflammatory and tolerogenic immune responses in AKI. **(A)** Renal ischaemia−reperfusion injury. **(B)** Cis-platinum induced tubular injury. **(C)** Sepsis-induced AKI.

RIRI is defined as a pathophysiological phenomenon in which there is a temporary loss of renal blood flow and tissue perfusion, followed by regaining of blood supply and increased tissue damage. RIRI is the leading cause of acute renal failure and transplant renal insufficiency. Early after ischemic injury, inflammatory mediators, including tumor necrosis factor-α (TNF-α), are produced, and previous studies have shown that TNF secretion is usually attributed to infiltrating monocytes, resident or infiltrating macrophages, and DCs (78). Subsequent studies have revealed that renal resident F4/80+ CD11c+ DCs have been shown to be the primary producers of TNF-α, and despite significant phenotypic overlap with resident macrophages, these cells migrate from the injured kidney to the draining lymph nodes within 24 to 48 hours of acute injury-a typical feature of DC function (79). After RIRI, DCs are activated and recruited to the kidneys, which mediates efficient induction and activation of adaptive immunity, antigen presentation to T cells and activation of T cells (mainly naïve antigen-specific CD8^+^ cells) (69). Hypoxia is an important marker of RIRI and regulates the innate immune response during this process (6). Previous studies have shown that DC activation in RIRI is accompanied by an increase in HIF-1α protein levels, and knockdown of HIF-1α significantly inhibits DC maturation and impairs the stimulation of allogeneic T cells (80, 81). In contrast, a recent study showed that HIF-2α deficiency in DCs upregulated CD36 expression in DCs, leading to cellular lipid accumulation, causing the overactivation of natural killer T (NKT) cells the production of IFN-γ and IL4, and ultimately exacerbating RIRI in mice (82). This finding suggests that hypoxia plays a crucial role in DC activation, and targeting and regulating HIF-1α or HIF-2α expression could be a potential therapeutic strategy for RIRI. The expression of miR-21 in hypoxia/reoxygenation-treated BMDCs significantly increased the proportion of mature DCs (CD11c^+^/MHC-II^+^/CD80^+^), and miR-21 overexpression could reduce the expression of HIF1α in RIRI and inhibit the maturation of DCs to protect epithelial cells from ischaemia reperfusion injury (83). Another study showed that miR-21 could target and regulate the CCR7 receptor on the surface of mature BMDCs and reduce the maturation of DCs and that RIRI-induced proinflammatory cytokine production could be attenuated by transferring miR-21-overexpressing BMDCs (84). During the transition from normal renal repair to maladaptive fibrosis in RIRI, persistent GM-CSF expression in renal tubular cells significantly increases monocyte chemoattractant protein-1 (MCP-1) expression in macrophages, which activates DCs and induces the secretion of the proinflammatory factors TNF-α and IL-1β by T cells. By inhibiting the expression of CCR2, which is a receptor for CCL2, the accumulation and persistence of macrophages, DCs, and T cells in the kidneys can be reduced, and therapeutic inhibition of CCL2/CCR2 signaling attenuates fibrosis and inflammation after RIRI (85). The regulatory mechanism by which kDCs affects RIRI is shown in **Figure 4A**.

### 5.2 Cis-platinum induced tubular injury

Renal tubular epithelial cells are particularly susceptible to pharmacotoxic injury and undergo necrotic apoptosis. In a prior investigation, it was demonstrated that in a cisplatin-induced acute tubular injury model, mice with DC depletion through the use of diphtheria toxin experienced increased renal dysfunction, tubular injury, neutrophil infiltration, and mortality compared to mice without DC depletion. Additionally, the authors provided evidence that the increase in renal injury could be attributed to the depletion of haematopoietic cells expressing CD11c (86). These studies show that renal DCs play a role in reducing cisplatin-induced kidney damage and the resulting inflammation. This protective mechanism is believed to be linked to the secretion of IL-10 by renal DCs immediately following cisplatin treatment (87, 88). However, it remains unknown whether activated DCs stimulate IL-10 secretion by Treg cells to protect against cisplatin-induced kidney injury. In contrast, pDCs express low levels of CD11c and are not targeted in these mouse models (89). Another study showed that renal tubular epithelial cells produce chemokines in response to IFN-α stimulation, and these chemokines initiate the recruitment and activation of pDCs, increasing the local production of IFN-α and leading to the development of renal interstitial inflammation and apoptosis in renal tubular epithelial cells (90). The regulatory mechanism by which kDCs affect cis-platinum-induced tubular injury is shown in **Figure 4B**.

### 5.3 Sepsis-associated AKI (SA-AKI)

AKI is a common form of tissue damage and organ dysfunction that occurs during sepsis (91). SA-AKI occurs mainly due to the release of PAMPs (e.g., LPS) and DAMPs from damaged cells and tissues, which dysregulate the immune system and lead to systemic and renal inflammation, complement activation, mitochondrial dysfunction, and metabolic reprogramming (92). In the presence of systemic inflammation or renal inflammation, kDCs receive inflammatory stimuli and increase their transfer and antigen-presenting capacity in the renal lymph nodes. In LPS-induced AKI, DCs in the renal interstitium were shown to migrate into the renal lymph nodes and stimulate local activation of CD4^+^ T cells and the production of IL-18, IFN-γ and TNF-α (93, 94). These studies confirm that under septic conditions, kDCs are subjected to inflammatory stimuli, become activated and migrate, thereby mediating immune responses and exacerbating renal injury (**Figure 4C**). Deficiency of kDCs inhibits LPS-induced tubular and interstitial injury in the acute phase of AKI but delays tissue repair in the recovery phase, suggesting that kDCs influence SA-AKI progression and renal repair (94). kDCs are involved in the development of SA-AKI, and it is crucial to clarify the exact mechanisms of renal injury and repair mediated by kDCs, including the signaling pathways involved, the effects on T-cell activation, and the secretion of relevant cytokines.

Numerous molecules and signaling pathways play important roles in DCs and are involved in the development and progression of SA-AKI. The induction of proinflammatory factors in DCs requires the involvement of TLR2 and TLR4, and specific intercellular adhesion molecule-3-grabbing non-integrin (SIGN) on DCs captures nonintegrin 1, which interacts with TLR4 to regulate the inflammatory response of renal tubular epithelial cells and is involved in the pathogenesis of AKI (95, 96). The IL-18 receptor signaling pathway plays an important role in DC- and CD4^+^ T-cell-mediated inflammatory responses, and inhibiting IL-18Rα in LPS-induced AKI reduces the mRNA expression of IL-18, IFN-γ, TNF, and IL-6 in the kidney (97). The spleen tyrosine kinase (Syk) signaling pathway has an important role in DCs and neutrophils in SA-AKI, and the inflammatory cascade during SA-AKI can be limited by inhibiting the Syk signaling pathway (98). Furthermore, Bruton’s tyrosine kinase (BTK) was activated in DCs, neutrophils, and B cells during the onset of SA-AKI, and renal function after AKI could be ameliorated by inhibiting the BTK signaling pathway (99).

### 5.4 COVID-19-associated AKI

AKI is a common complication of COVID-19, reported in more than a quarter of COVID-19 patients, and the mortality rate of COVID-19-associated AKI is higher in hospitalized patients than in those without renal involvement (100). The lack of renal recovery in COVID-19-associated AKI survivors compared to patients with other forms of AKI is of particular concern (101, 102). Both innate and adaptive immune responses play critical roles in the recognition and elimination of foreign pathogens. However, an excessive immune response during SARS-CoV-2 infection can lead to disease severity and associated complications in COVID-19 (103). Patients with poor COVID-19 progression develop dysregulation of cytokines and chemokines such as IL-2, IL-6, GM-CSF, CXCL10, CCL2, CCL7, CCL3 and TNF (104, 105). Decreased absolute numbers, activation, and function of CD4^+^ and CD8^+^ T cells were observed in patients with severe COVID-19, as evidenced by significantly lower levels of TCR expression, T-cell migration stimulator (DDP4), TCR signaling kinase, and MHC II molecules (106). Notably, depletion of pDCs (the main source of IFN-α) was observed in patients with COVID-19, which may have led to the inability of a subset of patients to effectively clear the virus from renal cells (107). Despite the insights into immune infiltration and activation of the innate and adaptive immune system in COVID-19-associated AKI, further studies are needed on the exact molecular mechanisms mediated by DCs in which they are involved, which may represent promising future specific therapeutic approaches for COVID-19-associated AKI.

## 6 DCs–targeted therapies in AKI: a promising therapeutic strategy

### 6.1 Induction of tolerogenic DCs (tolDCs)

tolDCs are a class of DCs with immature phenotypes and tolerance-inducing properties that do not induce antigen-specific immune responses but rather mediate immune tolerance (34). tolDCs express low levels of MHC and costimulatory molecules and can disrupt effector T-cell responses and induce regulatory T-cell proliferation and the production of immunosuppressive factors (e.g., IL-10), thereby inducing immune tolerance (108, 109). The plasticity of DCs allows for phenotypic modulation through tolerogenic transcriptional program modulation or proinflammatory transcription factor inhibition, and these modified DCs can induce therapeutic immunosuppression *in vivo* through direct interactions with T cells (110). The induction of tolDCs for the treatment of AKI has become a promising therapeutic strategy and is being actively promoted.

In **Table 3**, we summarize the available strategies and mechanisms for treating AKI by inducing tolDCs. DCs and NKT cells play a key role in the initiation of the immune response to RIRI in mice, and blocking DC-mediated NKT cell activation could be a novel therapeutic strategy for the prevention of AKI. Li et al. protected the kidney from RIRI by using the A2AR activator ATL313 to induce the production of tolDCs, which targeted and blocked DC-NKT interactions, inhibited NKT cell activation and reduced IFN-γ production (111). This study provides proof-of-principle for the use of pharmacologic approaches to induce the production of tolDCs to treat AKI. Sphingosine 1-phosphate (S1P), an important intracellular and extracellular signaling molecule, is a natural ligand of five G-protein-coupled receptors (S1P1, S1P2, S1P3, S1P4, and S1P5) that regulate cellular functions and modulate the immune system. S1P1 receptor agonist (FTY720) attenuated RIRI in mice, and *in vitro* studies showed that FTY720-treated DCs were rich in mitochondria, and further transplantation of FTY720-DCs observed that transfer of mitochondria-rich DCs protected the kidneys from RIRI because the transferred DCs donated their mitochondria to recipient splenocytes (i.e., macrophages), increased the activation of CD4FoxP3^+^ Tregs, and inhibited TNF-α production (112). In addition, in S1P3-deficient RIRI DCs exhibited reduced expression of costimulatory molecules, MHC II, and proinflammatory cytokines and chemokines and promoted Th2/IL-4 responses (113). This finding suggests that antagonizing S1P3 expression may result in the formation of tolDCs and could be used as a treatment for RIRI, but there are currently no studies of the use of S1P3 inhibitors to induce tolDCs. An mTOR inhibitor (rapamycin) reduced the immunogenicity and immunostimulatory phenotype of DCs after LPS stimulation, induced resistance to phenotypic maturation induced by proinflammatory stimuli, and modulated mitochondrial dynamics in DCs by increasing mitochondrial numbers, decreasing TNF-α and IL-6 secretion, and increasing IL-10 secretion, synergistically protecting the kidney from ischaemic injury (114, 115). Notably, in a recent study of vitamin D3/IL-10-conditioned tolDCs, treated cells exhibited high PD-L1 and CD86 expression, elevated IL-10 levels, decreased IL-12 p70 secretion, and suppressed transcriptome inflammatory profiles, which successfully abrogated renal injury without altering the infiltrating inflammatory cell population during systemic infusion (116). Current evidence from transplant-tolerant phase I/II clinical trials suggests that tolDCs are safe, and multiple regimens have been used to induce tolDC production, providing the basis for the clinical use of tolDC therapy in AKI. Further exploration of the role of tolDCs in AKI and postinjury repair is necessary in the future.

**Table 3:** Induction strategy and characterization of tolDCs for the treatment of AKI.

Induction strategy	Phenotype	Intervention	Model	Mechanism	Refs
Adenosine-2A receptor agonist (ATL313)	CD40^low^; OX40L^low^; B7-DC^high^	ATL313 (1 ng/kg, i.v.)	RIRI mouse	IFN-γ^+^ NKT cell↓	(111)
Sphingosine-1-phosphate agonist (FTY720)	CD80^low^; CD86^low^; MHCII^low^; IL-10^high^	1 μM FTY720 processing BMDC (0.5×10^6^, i.v.)	RIRI mouse	CD4FoxP3 Tregs↑, mitochondria↑, TNF-α↓	(112, 113)
mTOR inhibitor (Rapamycin)	CD80^low^; CD86^low^; CD40^low^; MHCII^low^; IL10^low^	10 ng/mL rapamycin processing BMDC (0.5×10^6^, i.v.)	RIRI mouse	TNF-α↓; IL-6↓, IL-10↑, mitochondria↑	(114, 115)
Vitamin-D3 and IL-10	CD86^low^; CD40^low^; MHCII^low^; PDL1^high^	20 nM VitD3 and 10 ng/ml IL-10 processing DC (1×10^6^, i.p.)	RIRI mouse	PDLl: CD 86↑, lymphocyte↓, IL-10↑ , IL-12 p70↓	(116)

"↑" indicates increased expression and "↓" indicates decreased expression.

### 6.2 Targeting cytokine production and co−stimulation

After maturation, DCs secrete cytokines to stimulate and activate T cells or act directly on damaged tissues, including pro-inflammatory factors such as IL-1, IL-6, IL-12, TNF, and IFN-α. miR-21 is a key inhibitory factor in the maturation of DCs, which can inhibit the maturation of DCs and decrease the secretion of IL-12, IL-6, and TNF-α, and attenuate the local inflammation (83). At the onset of AKI, pDCs rapidly infiltrated the kidneys and promoted renal injury through the production of IFN-α, whereas pDCs knockdown reduced the release of IFN-α, decreasing further renal injury (90). Upon binding of DCs surface antigenic peptide MHC complexes to T-cell surface TCRs, co-stimulatory substance molecules can provide positive signals that increase T-cell stimulation. DCs surface co-stimulatory molecules include CD80(B7-1), CD86(B7-2) and CD40 (117). The deletion of Rictor in DCs was observed in the AKI model to cause an increase in CD80 and CD86 on the surface of DCs, causing enhanced migration to the damaged kidney and greater tissue damage (118). Targeted modulation of Rictor expression in DCs in can attenuate further damage in AKI, but there are no studies of Rictor agonists in AKI. By targeting and regulating DCs maturation, secretory factor release and co-stimulatory molecules provides a new direction for the treatment of AKI.

## 7 Conclusions and perspectives

Immunoinflammatory mechanisms mediate the development of AKI, and the protective and injurious effects of DCs and their regulatory factors on AKI are receiving increasing attention. This paper provides a comprehensive review of the various subpopulations of DCs and their respective functions, as well as the molecular mechanisms involved in maintaining homeostasis and facilitating injury and repair in AKI. Additionally, the research progress on the use of tolDCs as a potential therapeutic strategy for AKI was discussed. The migration of immature DCs plays a crucial role in maintaining homeostasis and inducing peripheral tolerance. Conversely, the migration of activated and mature DCs to renal lymphoid organs in AKI is a significant characteristic of DC-mediated immunity. Therefore, targeting DC chemokines to modulate their migratory capacity has the potential to treat AKI. Additionally, therapeutic strategies to induce tolDCs by inhibiting proinflammatory cytokine release and costimulatory molecules are currently being developed. Promisingly, findings derived from animal studies indicate the efficacy of tolDCs in the treatment of AKI, and evidence from phase I/II clinical trials suggests a positive safety profile for tolDCs (119). Consequently, it is imperative to pursue additional research elucidating the intricate molecular mechanisms involved in the interaction between DCs, tolDCs, and other renal cells. These investigations can enhance our understanding of the underlying mechanisms driving immune inflammation-induced AKI and pave the way for novel therapeutic interventions.

## Author contributions

DL: Writing – original draft. HJ: Writing – review & editing. XY: Writing – review & editing. YL: Conceptualization, Writing – review & editing. WN: Conceptualization, Writing – review & editing. DZ: Conceptualization, Supervision, Writing – review & editing.
